# *In Silico* Prediction of the Mode of Action of *Viola odorata* in Diabetes

**DOI:** 10.1155/2020/2768403

**Published:** 2020-10-31

**Authors:** Manal Ali Buabeid, El-Shaimaa A. Arafa, Waseem Hassan, Ghulam Murtaza

**Affiliations:** ^1^College of Pharmacy and Health Sciences, Ajman University, Ajman 346, UAE; ^2^Department of Pharmacology and Toxicology, Faculty of Pharmacy, Beni-Suef University, Beni Suef 62514, Egypt; ^3^Department of Pharmacy, COMSATS University Islamabad, Lahore Campus, 54000, Pakistan

## Abstract

**Background:**

The metabolic syndrome increases the risk of different diseases such as type 2 diabetes. The prevalence of metabolic syndrome has rapidly grown and affected more than 230 million people worldwide. *Viola odorata* is a traditionally used plant for the treatment of diabetes; however, its mechanism to manage diabetes is still unknown.

**Purpose:**

This study was designed to systematically assess the mechanism of action of *Viola odorata* in diabetes.

**Methods:**

An extensive literature search was made to establish an ingredient-target database of *Viola odorata*. Of these, targets related to diabetes were identified and used to develop a protein-protein interaction network (PPIN) by utilizing the STITCH database. The obtained PPIN was assessed through Gene Ontology (GO) enrichment analysis based on ClueGO plugin.

**Results:**

According to the acquired data, there were about 143 chemical constituents present in *Viola odorata* having 119 protein targets. Of these, 31 targets were established to give the pharmacological effect against diabetes. The UniProt database was used for screening of 31 targets, out of which *Homo sapiens* contained 22 targets. Ultimately, 207 GO terms, grouped into 41 clusters, were found by gene analysis, and most of them were found to be linked with diabetes. According to findings, several proteins including TP53, BCL2, CDKN1A, 1L6, CCND1, CDKN2A, and RB1 have a significant role in the treatment of diabetes by *Viola odorata*.

**Conclusion:**

The possible activity of *Viola odorata* in the management of diabetes may be mediated by several molecular mechanisms, including the glutamine metabolic process, IRE1-mediated unfolded protein response, and pentose metabolic process.

## 1. Introduction

Metabolic syndrome is a group of interlinked diseases that occur together, for instance, hypertension and hyperglycemia, and leads to increased risk of type 2 diabetes. The prevalence of metabolic syndrome has rapidly grown. To date, it has affected more than 230 million people worldwide [1]. Obesity is one of the major contributing factors in producing metabolic syndrome. The major drawback of obesity is non-insulin-dependent diabetes mellitus and cardiovascular diseases [2]. Diabetes is a metabolic disorder categorized by hyperglycemia resulting from defects in insulin secretion, insulin action, or both [3]. According to a report based on death certificates conducted in 2010, it is the sixth leading cause of death in the US. In Pakistan, this disease is more prevalent in the urban population (11.1%), as compared to rural (9.4%) [4].

The medicines clinically used for the treatment of diabetes have many adverse effects, and over time, half of the drugs have become less responsive to a metabolic disorder. Thus, alternative therapy always remains the second option to treat diseases like metabolic disorder. It has already been reported that the *Viola odorata* plant is traditionally used to treat diabetes [5]. *Viola odorata* Linn. is a medicinal plant that belongs to the family Violaceae [5]. It is commonly known as sweet violet and found in Europe and Asia [6]. *Viola odorata* has been reported to contain phenolics, flavonoids, tannins, proteins, saponins, and alkaloids [7]. The active compounds present in *Viola* include salicylic acid, anthocyanins, butyl-2-ethylhexyl phthalate, and 5,6,7,7a-tetrahydro-4,4,7a-trimethyl-2(4H)-benzofuranone. Other active compounds of *Viola odorata* are cyclotides such as cycloviolacin O1, which are responsible for the antidiabetic potential of this plant. Approximately, 30 cyclotides have been recognized to date. Although there are many studies related to the antimetabolic effect of *Viola odorata* [5, 7, 8], there is no study that discusses the mechanism of action of *Viola odorata* as an antidiabetic agent.

Besides the “one drug-one target” model of drug discovery, “multiple drugs-multiple targets” concept, also known as polypharmacology, is widely spreading, especially in the field of multitargeted natural products and their mixtures including various nutraceuticals and traditional Chinese medicines (TCMs). This paradigm is promisingly elaborated by network pharmacology, which is a rapidly growing concept comprising integrated knowledge of polypharmacology and system biology [9]. Network pharmacology plays two essential roles in drug development. Firstly, it contributes to establishing a logical network model and predicting the drug targets based on the literature or publicly available databases. Secondly, the mode of drug action can be explored for the network equilibrium principle [10]. Network pharmacology is useful for developing a network comprising chemical ingredient-protein-disease and acquiring vital information about network protein regulation, which facilitates the drug discovery process through the knowledge of genome project and Gene Ontology (GO) [11–13]. In network pharmacology, molecular docking plays an essential role in the investigation of the mode of action of therapeutic moieties, particularly a multi-ingredient regimen [12].

The present study described the use of network pharmacology including molecular docking to predict the mode underlying the antidiabetic effects of *Viola odorata*. In this approach, chemical ingredients of *Viola odorata* were searched, ingredient-related protein targets were identified, and core functions were recognized through the protein-protein interaction network and molecular docking. To our knowledge, the mode of action of *Viola odorata* has not yet been studied by a network pharmacology approach; therefore, our approach offers a potential strategy to explore the mode of *Viola odorata* against diabetes.

## 2. Methodology

**Figure 1:**
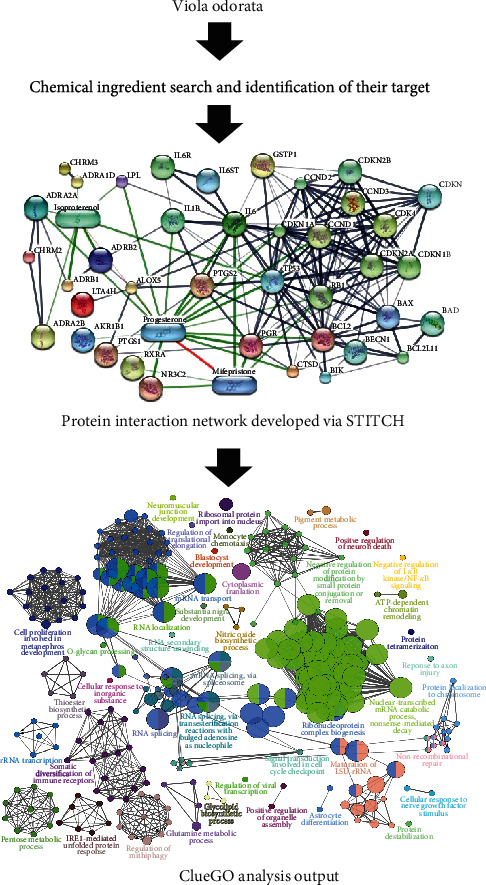
Flowchart demonstration of steps carried out in this systematic study.

In the present era, computational methods have become more useful to compile data related to drug discovery and for the identification of drug targets. The mechanism of *Viola odorata* against diabetes can be described by using the STITCH database along with network development and its investigation. This network development approach was aimed at finding out the multitarget compounds (Figure 1). At first, the chemical components of *Viola odorata* and their protein targets in *Homo sapiens* were defined. Secondly, a network was created called the *Viola odorata*-target interaction network, which was further assessed by different Gene Ontology (GO) terms. The molecular mechanism of *Viola odorata* against diabetes was explored through GO enrichment analysis and biological assessment utilizing Cytoscape and its plugin, ClueGO.

### 2.1. Recovery of Chemical Constituents and Their Targets

*Viola odorata* was searched for its chemical constituents using literature [12–20] as well as the Cybase database (http://www.cybase.org.au/index.php, accessed in November 2019). After retrieving the protein targets of these constituents, UniProtKB (http://www.uniprot.org, accessed in November 2019) was used for the extraction of gene names. To identify their association with diabetes, these molecular targets were examined based on the Kyoto Encyclopedia of Genes and Genomes (KEGG, http://www.kegg.jp/, accessed in November 2019).

### 2.2. Network Construction and Its Analysis

The mode of action of *Viola odorata* was described systematically by analyzing the interaction between identified targets by using the STITCH 5.0 database (http://stitch.embl.de/). This database gave us the statistics of predicted and known protein interactions, which were classified into direct and indirect interactions. The sources to find out the information of these interactions were high-throughput experimental findings, coexpression, genome studies, and text mining. The STITCH 5.0 database technique comprised the data of 9.6 million proteins extracted from approximately 2300 organisms. Then, the analysis of the association between the chemical ingredient and the identified protein targets was done by using the interaction network to assess the mode of action of *Viola odorata* and its pharmacodynamic components.

### 2.3. GO Enrichment Analysis

For dissection of the target genes in a hierarchically structured mode, GO enrichment analysis was introduced to identify and assess the specific biological properties of the potential targets. This approach is reasonably promising for the investigation of the mode of action of *Viola odorata* against diabetes. Cytoscape 3.4.0 software and its plugin ClueGO were used to develop, visualize, and evaluate the protein target network and assess the biological pathways to find out the mode of action of *Viola odorata* and its pharmacodynamic features [22, 23]. The *p* value threshold was set at 0.05 for ClueGO analysis. Additionally, this study involved the use of a medium network type option and a two-sided hypergeometric test with the Bonferroni correction. In comparison to the global and the detailed type of networks, the medium network shows GO terms originated in the level of 4-8, with a medium number of genes associated and a medium percentage of uploaded genes found. Lastly, an organic layout algorithm was used to visualize the functional network.

### 2.4. Structure Preparation and Molecular Docking

A wide array of compounds such quercetin, alrestatin, eugenol, coumarin, limonene, shikimic acid, stigmasterol, and rutin are present in *Viola odorata.* The traditional Chinese medicine systems pharmacology database (http://lsp.nwsuaf.edu.cn/tcmsp.php) revealed that tumor necrosis factor (TNF) and aldose reductase (AR) are the main targets of these compounds.

**Figure 2:**
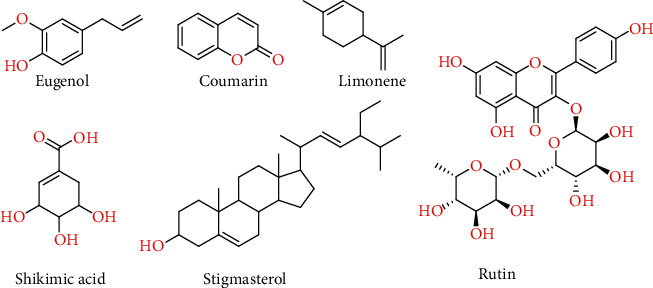
Chemical structures of the selected phytoconstituents for the molecular docking study.

3D structures of selected compounds eugenol, coumarin, limonene, shikimic acid, stigmasterol, and rutin (Figure 2) were constructed by the SYBYL-X 1.3/SKETCH module [24] followed by energy minimization according to the Tripos force field with the Gasteiger-Hücke atomic charge [24]. The cocrystallized structures of tumor necrosis factor (TNF) and aldose reductase (AR) were downloaded from RCSB Protein Data Bank (PDB ID: 2AZ5 [25] and 4GCA [26], respectively) to be regarded as starting points for molecular docking studies. The obtained 3D structures of 2AZ5 and 4GCA were carefully analyzed to authenticate the chemical accuracy using structure preparation tools in the biopolymer module of SYBYL-X 1.3 [5]. Missing hydrogen atoms were computationally added. Atom types were assigned appropriately, and atomic charges were applied according to the AMBER 7 ff99 force field. The SYBYL-X 1.3/protein preparation module [1] was then used to pick sensible protonation states for titratable residues, followed by energy minimization of each protein to avoid steric clashes using the Powell algorithm with a convergence gradient of 0.5 kcal/(mol·Å) for 1000 cycles, while keeping backbone atoms fixed. In order to investigate the binding modes of the selected compounds in the active site of proteins chosen, flexible molecular docking simulations were performed using the Surflex-Dock module of the molecular modeling software package SYBYL-X 1.3 [24] by adopting the same protocol and parameters as reported in our previous publications [27, 28]. For each ligand-receptor complex system, at least 20 best-docked poses were conclusively saved. The best putative poses of ligands were graded by adopting the Hammerhead scoring function (Cscore) [29]. Furthermore, the most frequently used TNF and AR inhibitors quercetin [30–34] and alrestatin [35–37], respectively, were used as benchmarks to compare docking poses of our tested compounds with those of standard compounds. Therefore, the standard compounds quercetin and alrestatin were also docked into corresponding target proteins TNF and AR, respectively, using the aforementioned protocol and parameters.

## 3. Results

### 3.1. Recovery of Chemical Ingredients and Their Targets

This work involved the retrieval of 143 chemical constituents of *Viola odorata* through a literature search (Supplementary Table 1). The features of these ingredients comply with the definition of a drug. Studies from different kinds of the literature confirmed that these chemical constituents had around 119 protein targets (Supplementary Table 2). The literature study reveals that *Viola odorata* is a useful alternative therapy for diabetes. Accordingly, out of 119, 31 targets of *Viola odorata* were found to exert pharmacological influence against diabetes. The UniProt database mapping (http://www.uniprot.org/) was used for the standardization of the identified 31 protein targets. Out of these 31 targets, 22 proteins were present in *Homo sapiens*.

### 3.2. Network Construction and Its Analysis

**Figure 3:**
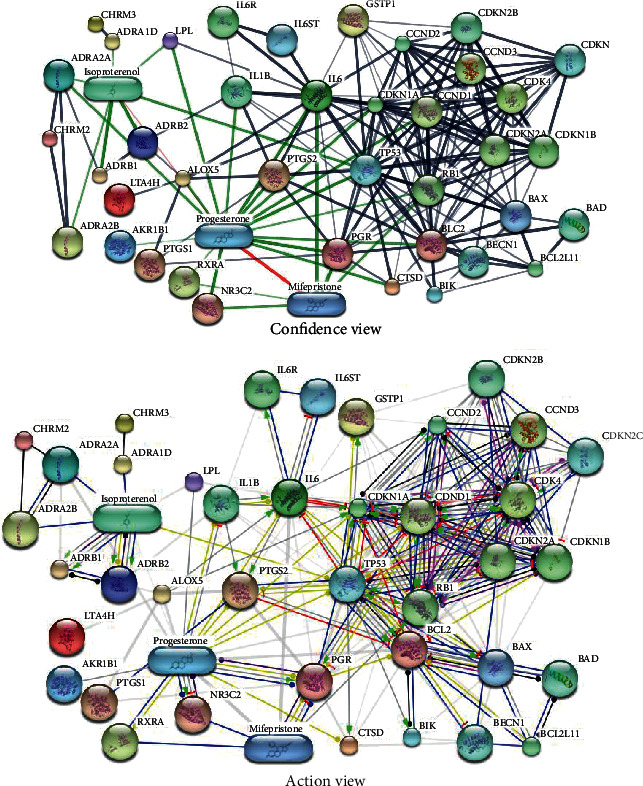
Protein-protein interaction network of diabetes-related protein targets of *Viola odorata*. In a confidence view, the nodes represent proteins. This network contains only colored nodes (no white node), indicating that the query proteins belong to the first shell of interactors only. Small nodes indicate proteins of unidentified 3D structure, while Large nodes reflect proteins with known 3D structure. The edges in this network represent protein-protein interactions, which are intended to be characteristic and expressive; i.e., proteins together play their role in a shared function; this does not essentially indicate that there is a physical attachment among them. Thick lines reflect stronger associations, while grey and green lines represent the protein-protein and chemical-protein associations, respectively. In an action view, the action type is shown by colored edges as follows: Activation, Inhibition, Binding, Catalysis, Phenotype, Posttranslational modification, Reaction, and Transcriptional regulation. Action effects are indicated by the following signs: Positive, Negative, and Unspecified. Note: LTA4H: leukotriene A4 hydrolase; CTSD: cathepsin D; CHRM3: cholinergic receptor, muscarinic 3; CDK4: cyclin-dependent kinase 4; IL6: interleukin 6 (interferon, beta 2); IL1B: interleukin 1, beta; ADRA2A: adrenoceptor alpha 2A; AKR1B1: aldo-keto reductase family 1, member B1 (aldose reductase); ADRB2: adrenoceptor beta 2, surface; LPL: lipoprotein lipase; CHRM2: cholinergic receptor, muscarinic 2; PGR: progesterone receptor; BCL2: B-cell CLL/lymphoma 2; NR3C2: nuclear receptor subfamily 3, group C, member 2; PTGS1: prostaglandin-endoperoxide synthase 1; PTGS2: prostaglandin-endoperoxide synthase 2; ADRB1: adrenoceptor beta 1; ALOX5: arachidonate 5-lipoxygenase; ADRA1D: adrenoceptor alpha 1D; GSTP1: glutathione S-transferase pi 1; ADRA2B: adrenoceptor alpha 2B; RXRA: retinoid X receptor, alpha; CCND1: cyclin D1; CCND3: cyclin D3; CDKN2A: cyclin-dependent kinase inhibitor 2A; RB1: retinoblastoma 1; CDKN1B: cyclin-dependent kinase inhibitor 1B; CDKN1A: cyclin-dependent kinase inhibitor 1A; IL6R: interleukin 6 receptor; BCL2L11: BCL2-like 11; CCND2: cyclin D2; CDKN2B: cyclin-dependent kinase inhibitor 2B (p15, which inhibits CDK4); BAD: BCL2-associated agonist of cell death; BECN1: beclin 1, autophagy related; CDKN2C: cyclin-dependent kinase inhibitor 2C; TP53: tumor protein p53; BIK: BCL2-interacting killer; IL6ST: interleukin 6 signal transducer; BAX: BCL2-associated X protein.

Twenty-two target proteins were present in the STITCH-based protein-protein interaction network (PPIN) (accessed in Nov 2019) (Figure 3). This PPIN was constructed using a medium probabilistic confidence score, i.e., 0.400. The network comprised 42 nodes and 152 edges. Network nodes act as the representatives of the protein targets or their relevant genes. The lines connecting various nodes indicate the interaction among the corresponding genes. There were 20 functional interactions out of 42 interactions. According to network statistics, the *p* value of PPIN enrichment was 0.001, which is a significantly very small value. It exhibits that there is a significant number of the observed edges, as well as the found nodes, which are not random. In the case of a random selection of nodes, the expected number of edges for this PPIN was 54. The average node degree of PPIN was 7.79, while its average clustering coefficient value was 0.749. In PPIN, the average number of interactions of protein targets at the threshold score is represented by the average node degree. Alternatively, the degree of connectivity of nodes in a PPIN is called the clustering coefficient. The connectivity of the network increases with the increased value of the clustering coefficient.

Moreover, 19 hubs were found in the PPIN. The node degree is a quantitative property of a node that can be explained as the number of interactions of nodes in a network. A node is known as a hub if its number of linkages is higher than the average node degree = 7.79. The findings revealed that BCL2 and TP53 proteins hold the highest node degree of the node, which is 23. Based on the node degree, IL6, CDKNIA, and progesterone were the subsequent proteins that had a degree of 18.  Table 1 shows the degree of each node. It has already been documented that diabetes has the involvement of these protein targets (in particular, the hubs and the functional nodes) such as TP53, BCL2, and CDKN1A.  Table 2 gives the action view of functional nodes present in PPIN, revealing that the activation of functional proteins except BIK, BAD, CCND1, and BCL2L11 could be done by *Viola odorata*. On the other hand, *Viola odorata* is involved in the inhibition of BIK, BECN1, IL6R, and BCL2L11. The results also describe the binding potential of *Viola odorata* ingredients with all identified functional proteins.  Table 2 also represents that *Viola odorata* is involved in many other functions such as cellular reactions, catalysis, posttranslational modifications, and gene expression.

### 3.3. GO Enrichment Analysis

**Figure 4:**
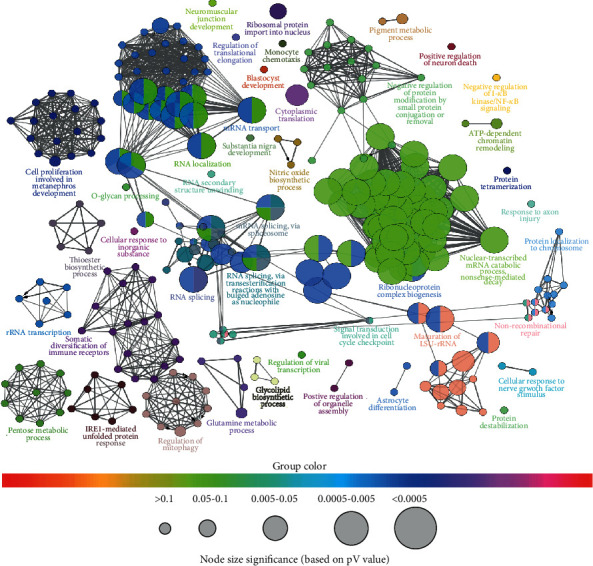
ClueGO-mediated retrieval of functionally grouped networks for the identified targets of *Viola odorata*. Each group is represented by the most significant term. The terms are represented by the nodes. The size of the nodes is associated with the significance of term enrichment. There is partial overlapping of the groups with similar functions.

ClueGO-mediated enrichment analysis was carried out for the identified protein targets of *Viola odorata* by using GO terms to explain their biological activities. As a result of this GO enrichment analysis, 41 GO terms associated with 207 genes were obtained in the form of small clusters, including the glutamine metabolic process, IRE1-mediated unfolded protein response, and pentose metabolic process (Supplementary Table 3, Figure 4). Further advancement on the mechanism of action of *Viola odorata* can be made based on the acquired findings.

### 3.4. Molecular Docking

In the present work, all six bioactive phytoconstituents were individually docked into the active site of TNF and AR proteins using the Surflex-Dock module of SYBYL-X 1.3. [38]. The docking scores (Cscore) of eugenol, coumarin, limonene, shikimic acid, stigmasterol, and rutin for TNF are 4.60, 2.03, 3.95, 2.58, 5.14, and 6.29, respectively, which indicates that rutin and stigmasterol exhibit substantial binding affinities towards TNF. The docking comparison with the standard compound has revealed that rutin and stigmasterol (Cscore = 6.29 and 5.14, respectively) share similar binding affinity towards TNF as that observed in the quercetin-TNF system (Cscore = 6.06). However, eugenol, coumarin, limonene, and shikimic acid displayed moderate to weak binding affinity towards TNF. Moreover, the docking of all six compounds along with standard compound alrestatin in the active site of AR protein has revealed that rutin and eugenol are more strongly bonded to AR (Cscore = 6.87 and 5.13, respectively) than alrestatin (Cscore = 4.94), whereas coumarin-, limonene-, shikimic acid-, and stigmasterol-bonded systems (Cscore = 3.41, 4.17, 2.04, and 4.58, respectively) have demonstrated moderate to weak binding affinity as compared to the alrestatin-AR system. As demonstrated by docking scores, rutin exhibits the highest binding affinity towards TNF and AR proteins among all selected and standard compounds, while shikimic acid has shown a nonsignificant binding affinity towards both proteins. However, the superior binding affinity of rutin to standard compounds towards TNF and AR might be attributed to its mighty structure substituted with various H-bond donor/acceptor features. For further details, corresponding docking scores and key residues involved in H-bonding are summarized in Supplementary Table 4.

**Figure 5:**
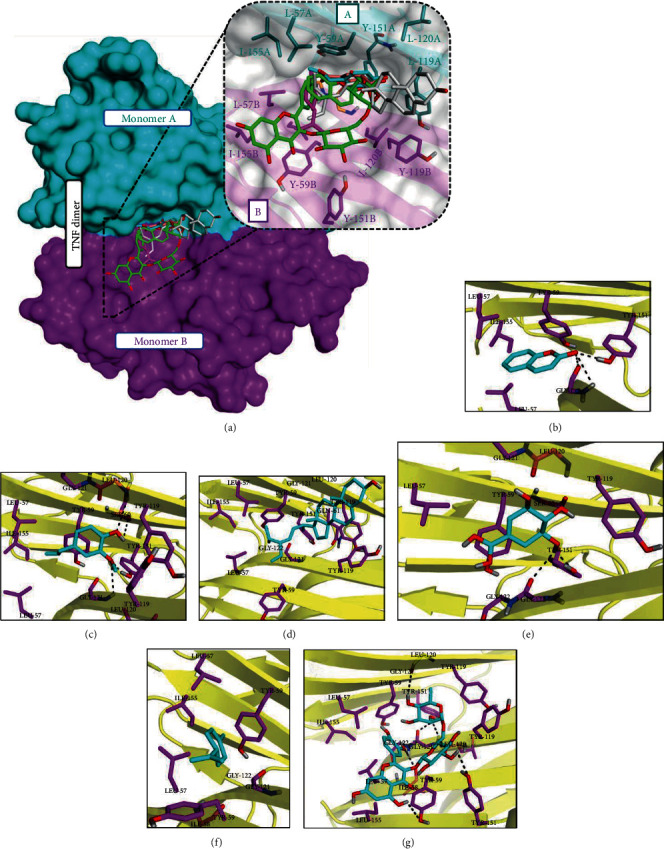
Obtained binding modes of ligands in the active site of tumor necrosis factor (TNF). (a) Docking conformations of the selected compounds bonded to the TNF dimer: (b) coumarin-TNF, (c) eugenol-TNF, (d) stigmasterol-TNF, (e) shikimic acid-TNF, (f) limonene-TNF, and (g) rutin-TNF.

To inspect the molecular features responsible for variations in binding affinity of selected ligands towards TNF, the top-ranked docking conformations for all six ligands in their respective complexes were saved. Docking results show that all ligands occupy the same cavity located between the subunits of the TNF dimer. As shown in Figures 5(b)–5(g), all ligands were able to establish at least a couple of H-bond interaction with the TNF dimer with the exception of limonene, which showed no H-bond interaction due to lack of any het-atom in its structure. Ideally, TNF-inhibitor interactions are hydrophobic and shape-driven, as the inhibitor structure needs to be large enough to interact with both subunits and to prevent binding of the third subunit to the TNF dimer [39]. Since nonpolar residues, i.e., Gly, Leu, and Tyr, are predominant in the binding site of the TNF dimer, it may explain good to moderate binding affinity of stigmasterol, eugenol, and limonene towards TNF (Figures 5(b), 5(d), and 5(f)). As shown in Supplementary Table 4, rutin forms the strongest complex with TNF as compared to all studied ligands. Graphical analysis of the rutin-TNF complex has demonstrated that the compound established six H-bond interactions with surrounding residues in a donor acceptor manner (Supplementary Table 4). Moreover, rutin exhibits large enough structure to interact with both subunits of the TNF dimer, which may be attributed to its higher binding affinity towards TNF than other studied complexes.

Conversely, the weakest binding affinity of coumarin and shikimic acid might be attributed to their smaller structure, which may not allow these structures to interact with both subunits of the TNF dimer. Analysis of quercetin-TNF docking pose reveals that the standard compound occupies the same cavity to establish at least six H-bond interactions with common residues as observed in the rutin-TNF complex (Supplementary Figures 1A and 1C). Superimposition of all docked complexes over the standard-TNF complex reveals that all ligands settled in the same binding site of TNF to establish similar pattern interactions.

**Figure 6:**
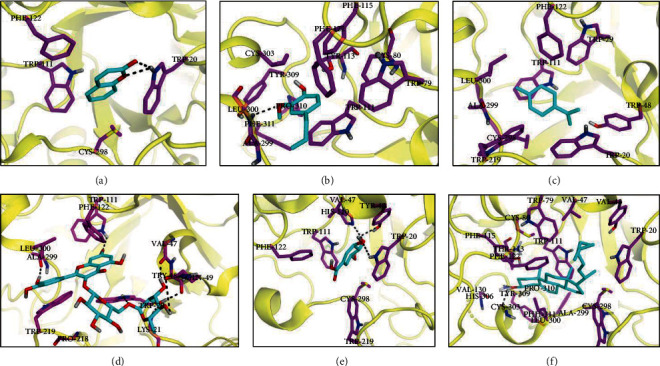
Obtained binding modes of ligands in the active site of aldose reductase (AR). (a) Docking conformations of the selected compounds bonded to AR: (b) coumarin-AR, (c) eugenol-AR, (d) stigmasterol-AR, (e) shikimic acid-AR, (f) limonene-AR, and (g) rutin-AR.

From Supplementary Table 4 and Figures 6(a)–6(f), out of the six compounds, rutin exhibited a high binding score and interaction towards AR. In the case of the rutin-AR complex (Figure 6(d)), the ligand interacted with the protein by forming seven hydrogen bonds, two *π*-*π* interactions, and several hydrophobic contacts. The two aromatic rings of rutin are firmly anchored in the anionic subsite by the formation of *π*-*π* interactions with Trp20 and Trp219. Furthermore, it forms hydrogen bonds with Trp20, Val47, Tyr48, Gln49, Trp111, and Ala299. In addition, residues Trp111, Phe122, Gln183, Pro218, and Leu300 are part of the specificity pocket to establish several hydrophobic and van der Waals (vdW) contacts.

Interestingly, these residues have previously been shown to be responsible for the selective ligand-protein complex formation [26]. Eugenol and stigmasterol have shown the second and third highest docking scores for AR as compared to the AR complex formation (Figures 6(a) and 6(f)). Although eugenol establishes only one H-bond interaction with Leu300, the aromatic ring of eugenol is packed between the top and bottom aromatic rings of residues Trp111 and Tyr309 to create *π*-*π* interactions with these residues. However, these additional *π*-*π* interactions were not observed in the stigmasterol-Ar complex, which may explain slightly higher docking scores for the eugenol-AR system. As demonstrated in Supplementary Table 4, coumarin and shikimic acid showed the least binding affinity towards AR proteins. Although coumarin and shikimic acid establish a greater number of H-bond interactions than the eugenol-AR system (Figures 6(a), 6(b), and 6(e)), the better binding affinity of eugenol to AR than coumarin and shikimic suggests that other nonpolar interactions also play a critical role apart from H-bonding interaction towards the binding of the ligand with the receptor. Like the TNF-bonded system, in the AR-standard complex, alrestatin establishes five H-bond interactions in the same binding cavity which was observed for all the studied compounds (Supplementary Figures 1B and 1D). Interestingly, a similar pattern of interaction has also been observed in the experimentally determined cocrystalized conformation of alrestatin in AR (PDB ID: 1AZ1). These findings further support the reliability of our docking results.

## 4. Discussion

*Viola odorata* is a medicinal herb used for centuries. This herb in the TCM system treats diabetes, bronchitis, and many kidney and liver disorders; however, the mechanism of action of this herb is still not known like other TCMs. So, in this study, an *in silico* analysis was done to find out the antimetabolic activity of *Viola odorata*. Thus, various interventions were adopted to conduct the *in silico* study of *Viola odorata*; these include the study of drug targets and building the protein-protein interaction network and pathways which were combined systematically to find out the potential mode of action. As a result of the protein target investigation, a total of 119 targets were achieved. These protein targets were analyzed by GO enrichment analysis, which further proved that *Viola odorata* had antimetabolic activity. According to pathway enrichment analysis, *Viola odorata* regulates many pathways in adjunct with a wide range of therapeutic modules.

According to literature, the active constituents of *Viola odorata* involved in the treatment of diabetes are aodoratine, rutin, limonene, coumarins, cycloviolacin, vanillic acid, eugenol, and stigmasterol [9–12]. These compounds exhibited their activity against different diseases in animal models. Aodoratine, in the form of chewed leaves, was used as an anticancer drug [14]. Coumarin is a potent antidiabetic agent, as it showed a decreased level of plasma glucose and increased level of insulin by activating insulin secretion via shutting K^+^ channels and opening Ca^++^ channels in rats [21]. Cycloviolacin O8, also known as nematocidal cyclotide, showed activity against prostate, breast, and ovarian cancer cell lines. Cycloviolacin showed anticancer activity due to its possible antiproliferative activity [22]. Eugenol has many uses as a pharmacological moiety. It is the main active constituent of *Viola odorata*. In an 5experiment, the fructose-fed rats were tested for the level of T-Ch, LDL-Ch, TG, and HDL-Ch for the treatment of diabetes. Resultantly, diabetes was promisingly treated due to antioxidant and lipid-lowering activity of eugenol [23]. The metabolic complications such as cardiovascular disease were also potentially treated by using the combination of aspirin and eugenol ester as compared to precursor aspirin due to lesser side effects [40]. It was reported that eugenol had potent anticancer activity against MCF-7 breast cancer cells through apoptosis and inhibition of cell division. The other activities involved are the reduction in glutathione level and an increase in the lipid peroxidation level [41]. Additionally, eugenol has strong activity against aflatoxin-induced lipid peroxidation. Aflatoxins, a carcinogenic agent, are produced as a secondary metabolite of *Aspergillus flavus*. The plant showed its effect by decreasing NADPH-dependent cytochrome C reductase [42]. The antidiabetic and antioxidative activity and insulin sensitivity of eugenol were studied by Al-Trad et al., which were reported in diabetic rats. Along with other mechanisms, the insulin sensitivity can be restored by the activation of the GLUT4-AMPK signaling pathway [43]. The antihyperglycemic activity was discussed in a study in which the liver marker enzymes, the enzymes of carbohydrate metabolism, i.e., creatinine kinase and urea nitrogen, were assessed after administration of eugenol to diabetic rats. The rats showed an improved marker value as compared to normal, in addition to an improvement in weight and hepatic values and reduced adverse effects [44]. Another constituent, limonene, has strong antimetabolic activity as an anticancerous and antidiabetic agent. d-Limonene showed activity as an antidiabetic agent by reducing the level of plasma glucose, glycosylated hemoglobin levels, and activity of gluconeogenic enzymes and increasing glucokinase activity and liver glycogen level [45]. It was confirmed that d-limonene not only decreases the white and brown adipocytes, serum triglycerides, and glucose levels in the blood of the obese mice but also inhibits lipid accumulation in the liver. It also reduces HDL and LDL via activation of the peroxisome proliferator-activated receptor involving *α*-signaling and inhibition of liver X-receptor by *β*-signaling [46]. Another research group reported the treatment of diabetes associated with nonalcoholic fatty liver disease by d-limonene [47]. In addition, the hypocholesterolemic effect of rutin was described, stating that rutin, in combination with lovastatin, decreases the level of LDL and HDL and liver enzymes [48].

In PPIN, the main hubs such as TP53, BCL2, CDKN1A, 1L6, CCND1, RB1, and CDNK2A have already been reported to be related to diabetes. TP53 is also known as a tumor suppressor gene. The downregulation of TP53 causes the development of cancer in humans. Downregulation and overexpression of TP53 in response to any external pressure may be associated with diabetes such as obesity and insulin resistance. In humans, the common TP53 polymorph expresses at codon 72, which produces protein variants R72 and P72. Thus, the R72TP53 is more active in fat deposition, nonalcoholic fatty liver disease, adipose tissue inflammation, and insulin resistance as compared to P721P53 [48]. According to another study, TP53 was not only found at the short arm of chromosome 17 but also linked with bladder cancer in humans; thus, it could be involved in tumorigenesis and can be used as a progression marker for the diagnostic purpose [49]. BCL2, another neurotrophic factor, is involved in regulating the primary function of the cell, viz., apoptosis. Any dysfunction in apoptosis causes various diseases, such as diabetes and cancer [50]. CDKN1A boosts up the cell division and cell cycle progression when targeted by an increased number of microRNA-93 in polycystic ovarian syndrome. The increased expression of microRNA-93 promotes cell division from the G1 to S phase. When the CDKN1A was eliminated from the granulosa cell, it promotes cell growth and cell cycle development from the previous phase. The reintroduction of CDK1A reverses its function, and instead of progression, it suppresses the growth and development of the cell. When insulin concentration is high in the cell, the microRNA-93 increases the sensitivity of the cell, which further activates KGN cell division and downregulation of CDK1A [51]. Interleukin-6 is a primary metabolic regulator and cytokine that plays a vital role in obesity and insulin resistance. It is a well-known fact that mitochondrial deformity is the reason for causing diabetes. Cells treated with IL6 have shown lesser lipids and a high level of glucose release rate; further, it also diminishes the ATP production and membrane potential and increases reactive oxygen species. Thus, it gives lipolytic effect and causes mitochondrial deformation, but it does not affect insulin sensitivity [52]. IL6 plays a crucial role in insulin resistance and obesity. The level of IL6 is increased in obese persons, revealing that IL6 is the major contributor to insulin resistance in diabetic conditions [53]. CCND1 is also known as cyclin D1 present throughout the cell cycle and involved in insulin signaling [54]. Rb1 is the active compound reported to induce adipogenesis and expression of peroxisome proliferator-activated receptor gamma. The PPARgamma has an antagonist called GW9662 that blocks Rb1-induced 3T3-L1 differentiation. The treatment results in decreased expression of miR-27b and pri-miR-27b. The antagonist of PPAR reduces the inhibitory effect of Rb1 on both miR-27b and pri-miR-27b. Thus, Rb1 acts through PPAR to decrease the expression of mir-27b and mature mir-27b that progresses adipogenesis and PPAR expression; thus, Rb1 has antidiabetic activity [55]. CDNK2A has already been documented to be associated with cancer and diabetes. It is a genomic locus that contains important genes for protein coding. The point at which antisense IncRNA is encoded is called ANRIL. Diabetes is induced when ANRIL gets polymorphed [56]. When the nutrition load increases, insulin secretion decreases, leading to the development of diabetes type 2. It is mainly caused by postprandial glucose regulation (PGR).

Pathway enrichment analysis reveals that most of the achieved GO terms have linkage with the diabetes. Nonetheless, three GO terms, i.e., glutamine metabolic process (GO: 0006536), IRE1-mediated unfolded protein response (GO: 0006986), and pentose metabolic process (GO: 0019321), are related to the pathogenesis of diabetes. The glutamine metabolic process represents the reactions and pathways involving the metabolism of glutamates. The diabetes-related conditions related to this GO term are hyperinsulinemia, hyperammonemia, and hypoglycemia [57]. Olivopontocerebellar atrophy is a common disease related to ataxia and Parkinson's-like diseases. The neurodegenerative process is linked with GDH (glycerate dehydrogenase) deficiency [58]. The regulation of diabetes is caused by genes including ASNS, CAD, GMPS, and PHGDH. Glutamate dehydrogenase secretes insulin from *β*-cells of the pancreas by activation of GDH via leucine [59]. When glutamate dehydrogenase enters the cell, glucose metabolism is activated via glucokinase resulting in the blockade of the K^+^ channel. This phenomenon is known as leucine-sensitive hypoglycemia [60]. On the other hand, diabetes is also caused by endoplasmic reticulum (ER) stress, which is caused by misfolding of proteins [61]. These diseases include obesity and type 2 diabetes mellitus. Other diseases caused by ER stress are atherosclerosis, cardiovascular disease, stroke, ischemia, NAFLD, alcoholic fatty liver disease, steatosis, and cirrhosis. Thus, the treatment of diabetes could be associated with the unfolded protein response. The IRE1-mediated unfolded protein response is a process that leads to a changed state or activity of a cell or an organism in the context of enzyme production/secretion or gene expression. IRE1 is the first and foremost signaling entity of unfolded protein response. There are two forms of IRE1, i.e., IRE1*α* and IRE1*β*. IRE1*α* is involved in signaling of unfolded protein response, and the IRE1*β* mechanism is still not clear. The genes involved in an unfolded protein response are ARFGAP1, SEC61A1, and TPP1. Under normal condition, when endoplasmic reticulum stress is not implied, IRE1*α* regulates the glucose protein [62]. When stress is applied by ER, IRE1*α* forms a homodimer and releases GRP78 which results in the interaction of the unfolded protein with ER resulting in obesity [63]. The unfolded protein response is an adaptive process that responds to metabolic stress, oxidative stress, and also inflammatory signaling pathways. For treatment purposes, the signaling of unfolded protein response is needed to be improved [64].

Pentose is a five-carbon sugar. The pentose metabolic process deals with the chemical reactions or pathways involving pentose. This pathway comprises two parts: an oxidative part and the nonoxidative part. The oxidative part is irreversible and allows the reduction of NADP to NADPH during the conversion of glucose-6-phosphate to pentose phosphate. While the nonoxidative part is reversible and interlinks the pentose phosphate to glycolytic intermediates [65], the genes found to be involved in the regulation of this pathway are DCXR, PGD, and PHGDH. The decreased level of pentose phosphate increases the occurrence of diabetes, likely due to the deficiency of G6PDH and RPI associated with sickle cell anemia [66]. It is also a documented fact that glucose-6-phosphate dehydrogenase is the main rate-limiting enzyme involved in the pentose phosphate pathway. It has a prominent function in controlling vascular functions and causing cardiovascular risks along with obesity and dyslipidemia diabetes. In hyperglycemia and obesity, the G6PD expression is increased, leading to the development of insulin resistance [67].

This study explains various mechanisms involved in the treatment of diabetes by using *Viola odorata* and explains that this plant enriches the identified target genes that are involved in the treatment of metabolic faults such as insulin resistance. In short, this study has evaluated the therapeutic mode of *Viola odorata* in diabetes in a preclinical manner, which provides a base in the field of evidence-based medicines.

## 5. Conclusion

In this study, the mechanism underlying the antidiabetic effects of *Viola odorata* is promisingly identified by network pharmacology and confirmed by molecular docking. The findings revealed that several constituents of *Viola odorata* such as rutin, eugenol, vanillic acid, stigmasterol, and limonene have an association with insulin activity, which suggests that *Viola odorata* has the potential to manage diabetes. The overall effect could be additive based on the concept of multichemical-multitarget. The findings of this *in silico* study have helped to predict the molecular mechanism of *Viola odorata* and provided a piece of confirmation of its clinical use against diabetes. Furthermore, molecular docking studies identified rutin, stigmasterol, and eugenol as the most potent TNF and AR inhibitors among our selected compounds when compared to their corresponding standard inhibitors quercetin and alrestatin, respectively. Graphical analysis has shown that the ligands capable of establishing various H-bonds as well as hydrophobic and van der Walls interactions are more strongly bonded in their corresponding complexes.

## Figures and Tables

**Table 1:** Node degree of diabetes-related protein targets of *Viola odorata* retrieved via STITCH.

Targets	Degree	Targets	Degree	Targets	Degree
LTA4H	1	PTGS1	3	IL6R	3
CTSD	6	PTGS2	11	BCL2L11	6
CHRM3	1	ADRB1	3	CCND2	14
CDK4	14	ALOX5	7	CDKN2B	12
IL6	18	ADRA1D	2	Isoproterenol	8
IL1B	8	GSTP1	8	BAD	3
ADRA2A	5	ADRA2B	3	BECN1	6
AKR1B1	1	RXRA	2	CDKN2C	11
ADRB2	4	CCND1	17	TP53	23
LPL	3	CCND3	11	BIK	4
CHRM2	2	CDKN2A	16	IL6ST	2
PGR	15	RB1	16	Progesterone	18
BCL2	23	CDKN1B	15	BAX	13
NR3C2	1	CDKN1A	18	Mifepristone	6

**Table 2:** Action view of predicted, functional protein targets (related to diabetes) of *Viola odorata* retrieved via STITCH.

Proteins	Activation	Inhibition	Binding	Phenotype	Catalysis	Posttranslational modification	Reaction	Expression	Score
CCND1	●	●	●				●	●	0.999
CCND3		●	●				●		0.999
CDKN2A	●	●	●		●	●			0.999
RB1	●	●	●		●	●	●		0.999
CDKN1B	●	●	●		●	●	●	●	0.999
CDKN1A	●	●	●		●	●	●	●	0.999
IL6R	●		●						0.999
BCL2L11			●					●	0.999
CCND2	●	●	●				●		0.999
CDKN2B	●	●	●		●	●			0.999
Isoproterenol	●	●	●		●			●	0.999
BAD		●	●				●		0.999
BECN1			●					●	0.999
CDKN2C	●	●	●		●	●			0.999
TP53	●	●	●			●	●	●	0.999
BIK			●				●		0.999
IL6ST	●	●	●						0.999
Progesterone	●	●	●		●			●	0.999
BAX	●	●	●			●		●	0.999
Mifepristone	●	●	●		●			●	0.999

## Data Availability

The supporting data has been provided as a supplementary file.

## Supplementary Materials

Supplementary Materials The chemical constituents of *Viola odorata* and their protein targets are available as Supplementary Tables 1 and 2. Supplementary Table 3 presents the Gene Ontology terms and their related genes retrieved through ClueGO. The Surflex score of docked ligands in the active site of tumor necrosis factor (TNF) and aldose reductase (AR) is given in Supplementary Table 4. Supplementary Figure 1 describes the obtained binding modes of standard compounds quercetin and alrestatin bonded to their target proteins TNF and AR, respectively.
