# Supplementary material for: In Silico Prediction of the Mode of Action of Viola odorata in Diabetes
Source: Biomed Res Int. 2020 Oct 31;2020:2768403. doi: 10.1155/2020/2768403 (PMC7803256; doi:10.1155/2020/2768403)
Supplement: Supplementary Materials — The chemical constituents of Viola odorata and their protein targets are available as Supplementary Tables 1 and 2. Supplementary Table 3 presents the Gene Ontology terms and their related genes retrieved through ClueGO. The Surflex score of docked ligands in the active site of tumor necrosis factor (TNF) and aldose reductase (AR) is given in Supplementary Table 4. Supplementary Figure 1 describes the obtained binding modes of standard compounds quercetin and alrestatin bonded to their target proteins TNF and AR, respectively. [file 2768403.f1.docx]

**Supplementary Table 1. Chemical constituents of *Viola* *odorata***

| **No.** | **Chemical Constituents** |
| --- | --- |
|  | (2E,6Z)-nona-2,6-dienol |
|  | (E, E)-hepta-2,4-dienal |
|  | Β-phenethyl formate |
|  | 1,8-ocimene |
|  | 10- Undecyn-1-ol |
|  | 1-ecosene |
|  | 1-Hexadecene |
|  | 1-Octadecene |
|  | 2, 3, 4- Trimethyl |
|  | 2,2,6,6-Tetramethyl-4-piperidinone |
|  | 2,4-Dimethyl dodecane |
|  | 2,5-heptadien-1-ol |
|  | 2-hexenal |
|  | 2-methoxy-3-(2-methyl propyl)pyrazine |
|  | 2-nitroproprionic acid |
|  | 2-pentyl-furan and ionone |
|  | 3,4-Dimethyl heptane |
|  | 3,5,5-trimethylcyclohex-2-enone |
|  | 3-hexenol |
|  | 3-pentadecenal |
|  | 4',5,7-Trihydroxy-6-methoxyisoflavone; 4',7-Di-O-b-Dglucopyranoside |
|  | 4-ethyl benzaldehyde, β-phenethyl formate |
|  | 5,10-pentadecadien-1-ol |
|  | 5,6,7,7a-tetrahydro-4,4,7a-trimethyl-2(4H)benzofuranone |
|  | Aodoratine |
|  | Benzofuranone |
|  | Butyl-2-ethylhexylphthalate |
|  | Circulin A |
|  | Cis-rose oxide |
|  | Citronellal |
|  | Coumarins |
|  | Cyclotide c2 precursor |
|  | Cyclotide c1 precursor |
|  | Cyclotide c3 precursor |
|  | Cyclotide k1 precursor |
|  | Cyanidin 3-glycosides, 3-O-[-L-Rhamnopyranosyl-(1→6)-β-D-glucopyranoside |
|  | Cycloviolacin |
|  | Cycloviolacin A |
|  | Cycloviolacin O1 |
|  | Cycloviolacin O2 |
|  | Cycloviolacin O3 |
|  | Cycloviolacin O4 |
|  | Cycloviolacin O5 |
|  | Cycloviolacin O6 |
|  | Cycloviolacin O7 |
|  | Cycloviolacin O8 |
|  | Cycloviolacin O9 |
|  | Cycloviolacin O10 |
|  | Cycloviolacin O11 |
|  | Cycloviolacin O12 |
|  | Cycloviolacin O13 |
|  | Cycloviolacin O14 |
|  | Cycloviolacin O15 |
|  | Cycloviolacin O16 |
|  | Cycloviolacin O17 |
|  | Cycloviolacin O18 |
|  | Cycloviolacin O19 |
|  | Cycloviolacin O20 |
|  | Cycloviolacin O21 |
|  | Cycloviolacin O22 |
|  | Cycloviolacin O23 |
|  | Cycloviolacin O24 |
|  | Cycloviolacin O25 |
|  | Cycloviolacin O26 |
|  | Cycloviolacin O27 |
|  | Cycloviolacin O28 |
|  | Cycloviolacin O29 |
|  | Cycloviolacin O30 |
|  | Cycloviolacin O31 |
|  | Cycloviolacin O32 |
|  | Cycloviolacin O33 |
|  | Cycloviolacin O34 |
|  | Cycloviolacin O35 |
|  | Cycloviolacin O36 |
|  | Cyclotide c2 precursor |
|  | Cyclotide c1 precursor |
|  | Cyclotide c3 precursor |
|  | Cyclotide k1 precursor |
|  | Dimethyldodecane |
|  | Dimethylheptane |
|  | Dodecanol |
|  | Ethyl hexanoate |
|  | Eugenol |
|  | Gaultherin |
|  | Geraniol |
|  | Glucopyranoside |
|  | Heneicosane |
|  | Hexadecane |
|  | Hexadecanoic acid |
|  | Hexanoic acid |
|  | Kalata B1 |
|  | Kalata B2 |
|  | Kalata S |
|  | Limonene |
|  | Linalool |
|  | Macrocyclic peptides |
|  | Methyl salicylate |
|  | Mucilage |
|  | Myrosin |
|  | N-Hexadecanoic acid |
|  | Odoratine |
|  | Pent-1-en-3-ol, 3-methyl but-2-enal, 2-methoxy-3-(1-methyl ethyl) pyrazine |
|  | Pentadeca-5,10-dien-1-ol |
|  | Pentadecanoic acid |
|  | Pentane 2,3,4-Trimethyl |
|  | Pinene |
|  | Rutin |
|  | Salicylaldehyde |
|  | Shikimic acid |
|  | Spathulenol |
|  | Stigmasterol |
|  | Tetrahydro-4,4,7a-trimethyl-2(4H)-benzofuranone |
|  | Trans-rose oxide |
|  | Tridecane |
|  | Undecanal |
|  | Vaby A |
|  | Vaby D |
|  | Vanillic acid |
|  | Varv A |
|  | Varv E |
|  | Varv F |
|  | Varv D |
|  | Vibi E |
|  | Vibi G |
|  | Vibi H |
|  | Violacin |
|  | Violacin A |
|  | Violacin A Precursor |
|  | Violanin |
|  | Violanthin |
|  | Violaquercetin |
|  | Violarutinm |
|  | Violin |
|  | Violutoside |
|  | Vitamin C |
|  | Vitri E |
|  | Vitri peptide A |
|  | Vitri peptide A;11-L-serine analogue |
|  | Vodo peptide N. |
|  | Vodo peptide M |
|  | VocC |
|  | VocC Precursor (partial) |
|  | Violacin A precursor |

**Supplementary Table 2. Protein targets of the *Viola* *odorata* compounds**

| No. | Proteins |
| --- | --- |
|  | 3-hydroxy-3-methylglutaryl-coenzyme A reductase |
|  | 5,6-dihydroxyindole-2-carboxylic acid oxidase |
|  | Acetylcholinesterase |
|  | Alcohol dehydrogenase 1B |
|  | Alcohol dehydrogenase 1C |
|  | Aldo-keto reductase family 1 member C1 |
|  | Aldose reductase |
|  | Alpha-1A adrenergic receptor |
|  | Alpha-1B adrenergic receptor |
|  | Alpha-1D adrenergic receptor |
|  | Alpha-2A adrenergic receptor |
|  | Alpha-2B adrenergic receptor |
|  | Alpha-2C adrenergic receptor |
|  | Amine oxidase [flavin-containing] A |
|  | Amine oxidase [flavin-containing] B |
|  | Antizyme inhibitor 1 |
|  | Apoptosis regulator BAX |
|  | Apoptosis regulator Bcl-2 |
|  | Arachidonate 5-lipoxygenase |
|  | Aryl hydrocarbon receptor |
|  | Bacillolysin |
|  | Bcl-2 homologous antagonist/killer |
|  | Beta-1 adrenergic receptor |
|  | Beta-2 adrenergic receptor |
|  | Beta-lactamase |
|  | C5a anaphylatoxin chemotactic receptor |
|  | Calcium-transporting ATPase type 2C member 1 |
|  | CAMP-dependent protein kinase inhibitor alpha |
|  | Canalicular multispecific organic anion transporter 1 |
|  | Caspase-3 |
|  | Catalase |
|  | Catalase isozyme 1 |
|  | Cathepsin D |
|  | Cell division protein kinase 4 |
|  | CGMP-inhibited 3',5'-cyclic phosphodiesterase A |
|  | Choline-phosphate cytidylyltransferase A |
|  | Chymotrypsinogen B |
|  | Collagen alpha-1(I) chain |
|  | Cytochrome P450 1A1 |
|  | Cytochrome P450 1B1 |
|  | Cytochrome P450 2A6 |
|  | Cytochrome P450 3A4 |
|  | Cytochrome P450-cam |
|  | DNA topoisomerase II |
|  | Dopamine D1 receptor |
|  | Ecto-NOX disulfide-thiol exchanger 2 |
|  | Ferrichrome-iron receptor |
|  | G1/S-specific cyclin-D1 |
|  | Gamma-aminobutyric acid receptor subunit alpha-1 |
|  | Gamma-aminobutyric-acid receptor alpha-2 subunit |
|  | Gamma-aminobutyric-acid receptor alpha-3 subunit |
|  | Gamma-aminobutyric-acid receptor alpha-5 subunit |
|  | Gamma-aminobutyric-acid receptor subunit alpha-6 |
|  | Gamma-glutamyltransferase 5 |
|  | Glutamate receptor 2 |
|  | Glutamate--cysteine ligase catalytic subunit |
|  | Glutathione S-transferase A2 |
|  | Glutathione S-transferase P |
|  | Hyaluronan synthase 2 |
|  | Hyaluronate lyase |
|  | Ig gamma-1 chain C region |
|  | Inositol-3-phosphate synthase 1 |
|  | Insulin |
|  | Integrin beta-2 |
|  | Interleukin-1 beta |
|  | Interleukin-10 |
|  | Interleukin-6 |
|  | Interleukin-8 |
|  | Lactase-phlorizin hydrolase |
|  | Leukotriene A-4 hydrolase |
|  | LIPOPROTEIN lipase |
|  | Low affinity immunoglobulin epsilon Fc receptor |
|  | Lysozyme |
|  | Mineralocorticoid receptor |
|  | Mitogen-activated protein kinase 3 |
|  | mRNA of PKA Catalytic Subunit C-alpha |
|  | Mucin-1 |
|  | Muscarinic acetylcholine receptor M1 |
|  | Muscarinic acetylcholine receptor M2 |
|  | Muscarinic acetylcholine receptor M3 |
|  | NADPH--cytochrome P450 reductase |
|  | Neuronal acetylcholine receptor protein, alpha-7 chain |
|  | Nicotinate-nucleotide--dimethylbenzimidazole phosphoribosyltransferase |
|  | Nitric-oxide synthase, endothelial |
|  | Nuclear receptor coactivator 1 |
|  | Nuclear receptor coactivator 2 |
|  | Olfactory receptor 1D2 |
|  | Pepsin A |
|  | Phosphatidylinositol-3,4,5-trisphosphate 3-phosphatase and dual-specificity protein phosphatase PTEN |
|  | Probable E3 ubiquitin-protein ligase HERC5 |
|  | Progesterone receptor |
|  | Progesterone receptor |
|  | Prostaglandin G/H synthase 1 |
|  | Prostaglandin G/H synthase 2 |
|  | Protein kinase C beta type |
|  | Putative beta-glucuronidase-like protein SMA3 |
|  | Quinone oxidoreductase |
|  | Retinoic acid receptor RXR-alpha |
|  | Rhodopsin |
|  | Serine/threonine-protein phosphatase 2B catalytic subunit alpha isoform |
|  | Short transient receptor potential channel 3 |
|  | Sodium channel protein type 5 subunit alpha |
|  | Sodium-dependent dopamine transporter |
|  | Sodium-dependent noradrenaline transporter |
|  | Sodium-dependent serotonin transporter |
|  | Solute carrier family 22 member 5 |
|  | Sucrase-isomaltase, intestinal |
|  | Superoxide dismutase [Cu-Zn] |
|  | Thermolysin |
|  | Thrombin |
|  | Thromboxane A2 receptor |
|  | T-lymphocyte activation antigen CD86 |
|  | Transcription factor p65 |
|  | Transient receptor potential cation channel subfamily V member 3 |
|  | Trypsin-3 |
|  | Tumor necrosis factor |
|  | Type I iodothyronine deiodinase |
|  | Urokinase-type plasminogen activator |
|  | Xanthine dehydrogenase/oxidase |

Supplementary Table 3. Retrieval of GO terms and their related genes via ClueGO

| GO ID | GO Term | Term P-Value (¤) | Group P-Value (¤) | Associated Genes Found |
| --- | --- | --- | --- | --- |
| 21762 | substantia nigra development | 47.0E-3 (800.0E-3) | 47.0E-3 (700.0E-3) | [CKB, H2BFS, YWHAE] |
| 46782 | regulation of viral transcription | 69.0E-3 (550.0E-3) | 69.0E-3 (620.0E-3) | [NELFCD, SMARCB1, TRIM21] |
| 48678 | response to axon injury | 21.0E-3 (940.0E-3) | 21.0E-3 (430.0E-3) | [LAMB2, LGALS1, NAIP, TSPO] |
| 51262 | protein tetramerization | 10.0E-3 (710.0E-3) | 10.0E-3 (250.0E-3) | [ACACA, ANXA2, ASL, DCXR, DHRS4, RPS19] |
| 71241 | cellular response to inorganic substance | 9.2E-3 (670.0E-3) | 9.2E-3 (230.0E-3) | [BLM, CLU, EIF2S1, EIF4A3, FUS, TRAF2, TSPO] |
| 01824 | blastocyst development | 72.0E-3 (500.0E-3) | 72.0E-3 (570.0E-3) | [PRPF19, RPL7L1, SMARCB1] |
| 2181 | cytoplasmic translation | 19.0E-6 (2.5E-3) | 19.0E-6 (670.0E-6) | [RPL15, RPL26L1, RPL29, RPL36, RPL36AL, RPL7L1, RPL8] |
| 1901216 | positive regulation of neuron death | 74.0E-3 (440.0E-3) | 74.0E-3 (520.0E-3) | [CLU, EIF2S1, UBE2M] |
| 2548 | monocyte chemotaxis | 62.0E-3 (620.0E-3) | 62.0E-3 (620.0E-3) | [CCL17, RPL10, RPS19] |
| 6448 | regulation of translational elongation | 7.0E-3 (570.0E-3) | 7.0E-3 (190.0E-3) | [EIF4A3, RPS5, RPS9] |
| 6610 | ribosomal protein import into nucleus | 310.0E-6 (39.0E-3) | 310.0E-6 (10.0E-3) | [IPO4, RPL17, RPL23] |
| 7528 | neuromuscular junction development | 24.0E-3 (970.0E-3) | 24.0E-3 (460.0E-3) | [AGRN, LAMB2, UTRN] |
| 10501 | RNA secondary structure unwinding | 3.7E-3 (360.0E-3) | 3.7E-3 (110.0E-3) | [DDX19B, DDX21, DDX23, EIF4A3] |
| 16266 | O-glycan processing | 60.0E-3 (660.0E-3) | 60.0E-3 (660.0E-3) | [MUC3A, MUC5AC, ST3GAL3] |
| 31648 | protein destabilization | 15.0E-3 (870.0E-3) | 15.0E-3 (330.0E-3) | [NOMO2, SIRT6, TRIM21] |
| 43124 | negative regulation of I-kappaB kinase/NF-kappaB signaling | 53.0E-3 (690.0E-3) | 53.0E-3 (690.0E-3) | [RPL10, STAT1, USP10] |
| 43044 | ATP-dependent chromatin remodeling | 90.0E-3 (90.0E-3) | 120.0E-3 (360.0E-3) | [ACTL6A, MBD2, SMARCB1] |
| 42440 | pigment metabolic process | 90.0E-3 (90.0E-3) | 90.0E-3 (450.0E-3) | [FECH, GMPS, TSPO] |
| 48708 | astrocyte differentiation | 74.0E-3 (440.0E-3) | 29.0E-3 (530.0E-3) | [LAMB2, PRPF19, VIM] |
| 1902117 | positive regulation of organelle assembly | 53.0E-3 (690.0E-3) | 77.0E-3 (460.0E-3) | [CAPG, CNOT1, NCAPG] |
| 1990090 | cellular response to nerve growth factor stimulus | 14.0E-3 (850.0E-3) | 17.0E-3 (370.0E-3) | [BPTF, EIF4A3, RPL8] |
| 6809 | nitric oxide biosynthetic process | 87.0E-3 (170.0E-3) | 130.0E-3 (260.0E-3) | [CLU, IL1B, TSPO] |
| 6541 | glutamine metabolic process | 610.0E-6 (74.0E-3) | 49.0E-3 (690.0E-3) | [ASNS, CAD, GMPS, PHGDH] |
| 35384 | thioester biosynthetic process | 77.0E-3 (380.0E-3) | 110.0E-3 (460.0E-3) | [ACACA, ACLY, FAR1] |
| 09303 | rRNA transcription | 1.0E-3 (120.0E-3) | 3.3E-3 (100.0E-3) | [GTF3C3, GTF3C5, NCL, SMARCB1] |
| 726 | non-recombinational repair | 74.0E-3 (440.0E-3) | 5.5E-3 (160.0E-3) | [PAXIP1, PRPF19, RIF1] |
| 36498 | IRE1-mediated unfolded protein response | 77.0E-3 (380.0E-3) | 9.0E-3 (230.0E-3) | [ARFGAP1, SEC61A1, TPP1] |
| 377 | RNA splicing, via transesterification reactions with bulged adenosine as nucleophile | 2.1E-6 (300.0E-6) | 780.0E-9 (28.0E-6) | [DDX23, EIF4A3, FUS, GEMIN4, HNRNPA2B1, HNRNPR, PCF11, PRPF19, SF3B2, SMN1, SNRPA, SNRPD1, SNRPD2, SNRPE, SRSF11, U2AF1, USP39] |
| 398 | mRNA splicing, via spliceosome | 2.1E-6 (300.0E-6) | 780.0E-9 (28.0E-6) | [DDX23, EIF4A3, FUS, GEMIN4, HNRNPA2B1, HNRNPR, PCF11, PRPF19, SF3B2, SMN1, SNRPA, SNRPD1, SNRPD2, SNRPE, SRSF11, U2AF1, USP39] |
| 72395 | signal transduction involved in cell cycle checkpoint | 74.0E-3 (440.0E-3) | 3.1E-3 (100.0E-3) | [CNOT1, PRPF19, RPS27A] |
| 19321 | pentose metabolic process | 1.6E-3 (180.0E-3) | 46.0E-3 (750.0E-3) | [DCXR, PGD, PHGDH] |
| 70585 | protein localization to mitochondrion | 2.8E-3 (290.0E-3) | 6.7E-3 (190.0E-3) | [ACTL6A, MAGI1, POLR3A, RPL28, SLC25A6, TSPO, U2AF1, YWHAE, YWHAG] |
| 1903146 | regulation of mitophagy | 630.0E-6 (75.0E-3) | 6.7E-3 (190.0E-3) | [ACTL6A, POLR3A, RPL28, TSPO, U2AF1] |
| 470 | maturation of LSU-rRNA | 55.0E-9 (8.5E-6) | 90.0E-12 (3.7E-9) | [FTSJ3, NOL9, NSA2, RPF1, RPL35, RPL7A, RPL7L1] |
| 288 | nuclear-transcribed mRNA catabolic process, deadenylation-dependent decay | 90.0E-3 (90.0E-3) | 47.0E-9 (1.8E-6) | [CNOT1, EIF4A3, EXOSC3] |
| 72203 | cell proliferation involved in metanephros development | 560.0E-6 (68.0E-3) | 190.0E-3 (190.0E-3) | [MYC, OXSR1, STAT1] |
| 1903321 | negative regulation of protein modification by small protein conjugation or removal | 4.4E-3 (420.0E-3) | 3.7E-3 (110.0E-3) | [CDC26, PSMD13, PSMD2, RPS27A, RPS3, TRIM21, TSPO] |
| 2200 | somatic diversification of immune receptors | 85.0E-3 (250.0E-3) | 55.0E-3 (670.0E-3) | [EXOSC3, PAXIP1, RAG1] |
| 6403 | RNA localization | 100.0E-9 (15.0E-6) | 1.4E-9 (58.0E-9) | [ATR, CCT4, DDX19B, EIF4A3, EXOSC3, HNRNPA2B1, MVP, MYO1C, NUP153, NUP205, NUP37, RAE1, SEC13, SRSF11, U2AF1, UPF2] |
| 51028 | mRNA transport | 280.0E-9 (42.0E-6) | 1.4E-9 (58.0E-9) | [DDX19B, EIF4A3, HNRNPA2B1, MVP, MYO1C, NUP153, NUP205, NUP37, RAE1, SEC13, SRSF11, U2AF1, UPF2] |
| 22613 | ribonucleoprotein complex biogenesis | 6.8E-42 (1.4E-39) | 10.0E-36 (440.0E-36) | [ATR, BMS1, CNOT1, DDX21, DDX23 |

^¤^ Corrected with Bonferroni step down.

^≠^ DHX37, EIF2S1, EIF3E, EIF3FP2, EIF4A3, EXOSC3, FTSJ3, GEMIN4, HEATR1, MDN1, NOL9, NOP16, NSA2, POP4, PRPF19, RPF1, RPL10, RPL15, RPL17, RPL18, RPL19, RPL23, RPL26L1, RPL28, RPL29, RPL3, RPL30, RPL32, RPL35, RPL36, RPL36AL, RPL37A, RPL5, RPL7A, RPL7L1, RPL8, RPS13, RPS16, RPS18, RPS19, RPS21, RPS23, RPS25, RPS26, RPS27A, RPS3, RPS3A, RPS5, RPS6, RPS7, RPS9, SMN1, SNRPD1, SNRPD2, SNRPE, URB2, USP39, WDR36.

Supplementary Table 4**.** Surflex score of docked ligands in the active site of tumor necrosis factor (TNF) and Aldose reductase (AR).

| **Protein** | **Docking complex** | **CScore*^a^*** | **Crash score*^b^*** | **Polar score*^c^*** | **G score*^d^*** | **PMF score*^e^*** | **D score*^f^*** | **Chem score*^g^*** | **Amino acid interaction** |
| --- | --- | --- | --- | --- | --- | --- | --- | --- | --- |
| **Tumor necrosis factor (TNF)** | Quercetin | 6.06 | -0.75 | 2.18 | -79.70 | 4.45 | -99.70 | -16.134 | Q61, S60, Y119,  L120, Y151 |
|  | Eugenol | 4.60 | -3.76 | 2.60 | -155.47 | 22.90 | -181.65 | -14.52 | S60, Y119, G121, Y151 |
|  | Coumarin | 2.03 | -0.22 | 1.12 | -47.96 | 0.61 | -82.47 | -14.78 | G121, Y151 |
|  | Limonene | 3.95 | -1.06 | 1.13 | -138.97 | 24.03 | -60.83 | -16.03 | - |
|  | Shikimic acid | 2.58 | -1.33 | 2.25 | -118.106 | 1.25 | -59.24 | -6.92 | G121, Y151 |
|  | Stigmasterol | 5.14 | -0.79 | 1.71 | -129.34 | 7.14 | --76.061 | -16.91 | - |
|  | Rutin | 6.29 | -1.57 | 0.01 | -266.05 | 34.29 | -115.95 | -31.74 | I58A, Y59A, L120B, G121A, G122A, Y151A, Y151B |
| **Aldose reductase (AR)** | Alrestatin | 4.94 | -1.18 | 3.12 | -91.99 | 61.11 | -76.88 | -23.12 | R217, V297, C298, A299 |
|  | Eugenol | 5.13 | -0.85 | 1.11 | -167.72 | 5.58 | -89.69 | -22.79 | L300 |
|  | Coumarin | 3.41 | -0.26 | 0.97 | -114.81 | 4.88 | -68.24 | -20.74 | W20 |
|  | Limonene | 4.17 | -0.85 | 0.00 | -165.57 | 22.85 | -76.42 | -22.91 | - |
|  | Shikimic acid | 2.04 | -1.61 | 2.95 | -116.30 | 1.05 | -70.530 | -11.46 | W20, Y48, H110 |
|  | Stigmasterol | 4.58 | -2.67 | 0.00 | -270.30 | 21.36 | -120.33 | -33.44 | C303, Y309 |
|  | Rutin | 6.87 | -2.03 | 4.37 | -188.19 | -30.53 | -145.57 | -11.60 | W20, V47, Y48, Q49, W111, A299 |

***^a^*CScore** is a consensus scoring which uses multiple types of scoring functions to rank the affinity of ligands, ***^b^*Crash**-score revealing the inappropriate penetration into the binding site, ***^c^* Polar** region of the ligand, ***^d^* G-score** showing hydrogen bonding, complex (ligand-protein), and internal (ligand-ligand) energies, ***^e^* PMF-score** indicating the Helmholtz free energies of interactions for protein-ligand atom pairs (Potential of Mean Force, PMF), ***^f^* D-score** for charge and van der Waals interactions between the protein and the ligand, ***^g^* Chem-score** points for hydrogen bonding, lipophilic contact, and rotational entropy, along with an intercept term.


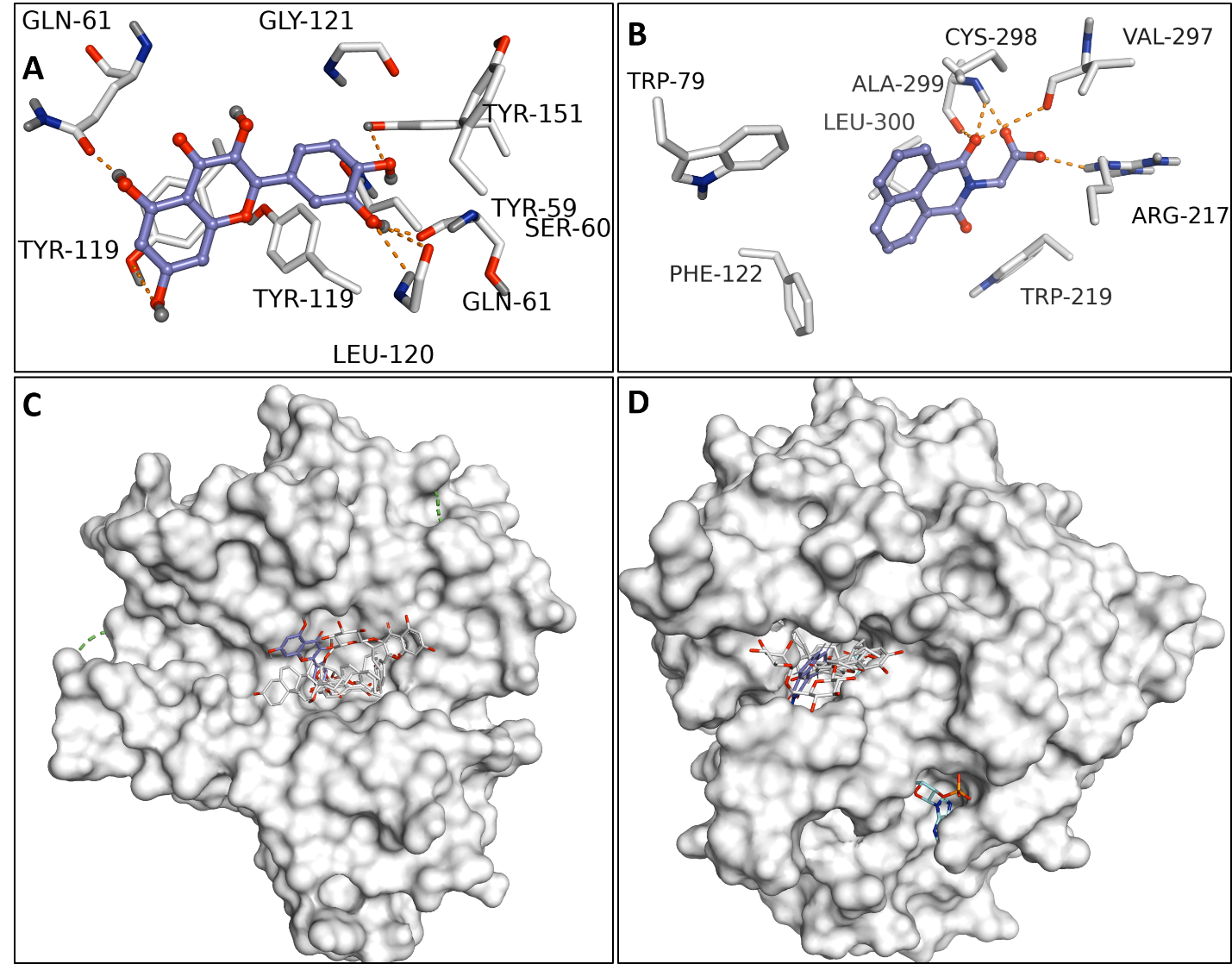


**Supplementary Figure 1.** Obtained binding modes of standard compounds quercetin and alrestatin bonded to their rtarget proteins tumour necrosis factor (TNF) and Aldose reductase (AR), respectively: (A) TNF-quercetin; (B) AR-alrestatin. (C) Comparison of binding mode of standard compound quersutin (slate) with corresponding ligands bonded to TNF. (D) Superposition of docking pose of standard compound alrestatin (slate) with corresponding ligands bonded to AR.
